# Supplementary figures and images for: Ion Separation Together with Water Purification via a New Type of Nanotube: A Molecular Dynamics Study
Source: Int J Mol Sci. 2023 Apr 3;24(7):6677. doi: 10.3390/ijms24076677 (PMC10094855; doi:10.3390/ijms24076677)

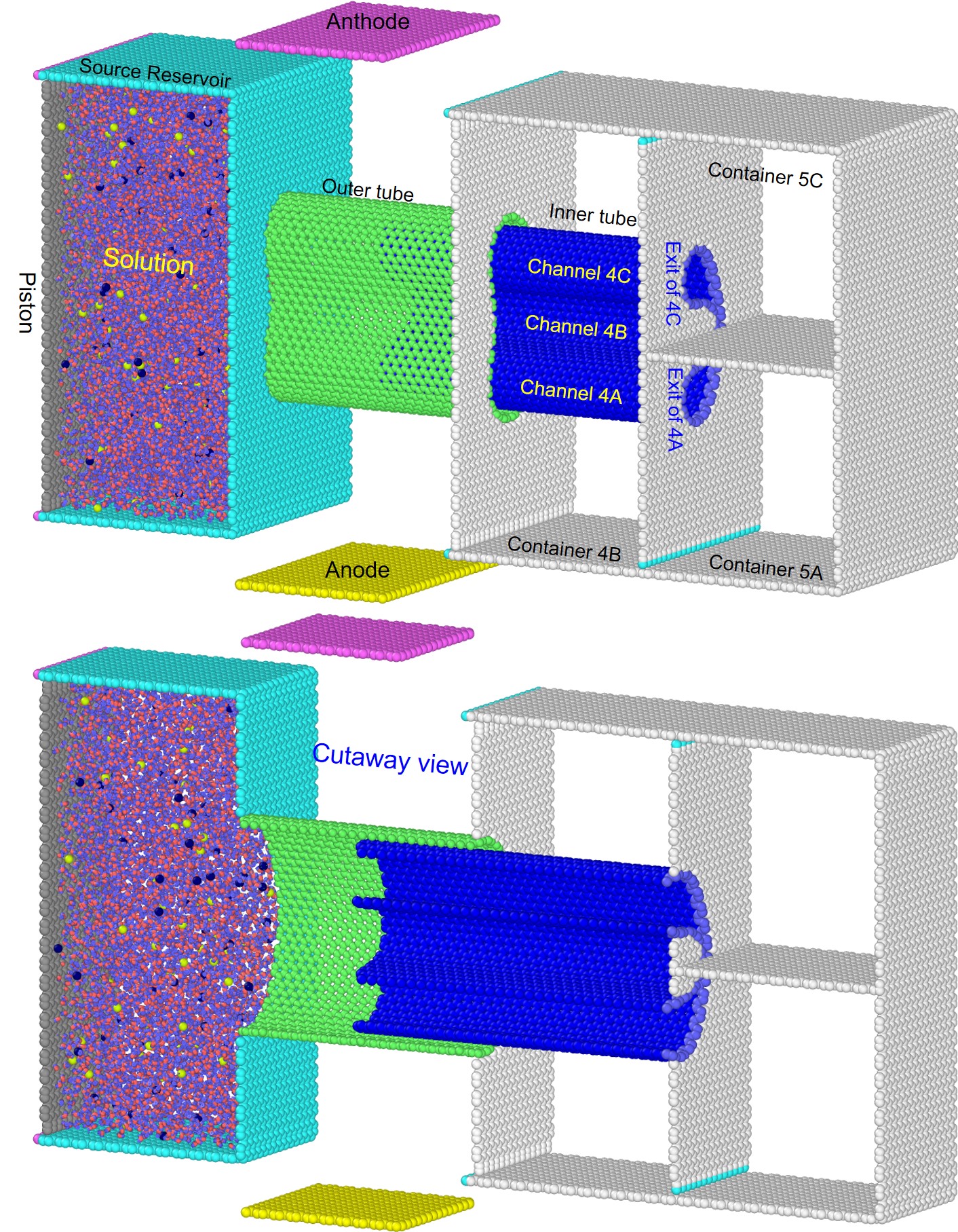

Supplement: Supplementary file 1 [file ijms-24-06677-s001.zip › The 3D geomtry of the present model.jpg]
